# Paratope Duality and Gullying are Among the Atypical Recognition Mechanisms Used by a Trio of Nanobodies to Differentiate Ebolavirus Nucleoproteins

**DOI:** 10.1016/j.jmb.2019.10.005

**Published:** 2019-10-15

**Authors:** Laura Jo Sherwood, Alexander Bryan Taylor, Peter John Hart, Andrew Hayhurst

**Affiliations:** 1- **Disease Intervention and Prevention,** Texas Biomedical Research Institute, San Antonio, TX, 78227, USA; 2- **X-ray Crystallography Core Laboratory,** Institutional Research Cores and Department of Biochemistry and Structural Biology, University of Texas Health Science Center at San Antonio, San Antonio, TX, 78229, USA; 3- **Department of Veterans Affairs,** South Texas Veterans Health Care System, San Antonio, TX, 78229, USA

**Keywords:** Antibody–antigen recognition, Nanobody, Filovirus, Restructuring, *Sudan ebolavirus*

## Abstract

We had previously shown that three anti–Marburg virus nanobodies (VHH or single-domain antibody [sdAb]) targeted a cryptotope within an alpha-helical assembly at the nucleoprotein (NP) C-terminus that was conserved through half a century of viral evolution. Here, we wished to determine whether an anti–Ebola virus sdAb, that was cross-reactive within the *Ebolavirus* genus, recognized a similar structural feature upstream of the ebolavirus NP C-terminus. In addition, we sought to determine whether the specificities of a less cross-reactive anti–*Zaire ebolavirus* sdAb and a totally specific anti–*Sudan ebolavirus* sdAb were the result of exclusion from this region. Binding and X-ray crystallographic studies revealed that the primary determinant of cross-reactivity did indeed appear to be a preference for the helical feature. Specificity, in the case of the *Zaire ebolavirus*–specific sdAb, arose from the footprint shifting away from the helices to engage more variable residues. While both sdAbs used CDRs, they also had atypical side-on approaches, with framework 2 helping to accommodate parts of the epitope in sizeable paratope gullies. The *Sudan ebolavirus*–specific sdAb was more remarkable and appeared to bind two C-terminal domains simultaneously *via* nonoverlapping epitopes—“paratope duality.” One mode involved paratope gullying, whereas the other involved only CDRs, with CDR3 restructuring to wedge in between opposing walls of an interdomain crevice. The varied routes used by sdAbs to engage antigens discovered here deepen our appreciation of the small scaffold’s architectural versatility and also reveal lucrative opportunities within the ebolavirus NP C-termini that might be leveraged for diagnostics and novel therapeutic targeting.

## Introduction

Filoviruses of the genera *Marburgvirus* and *Ebolavirus* continue to re-emerge in Africa, causing outbreaks of transmissible hemorrhagic fever with high human case fatality rates. *Marburgvirus* currently has one species known to cause human disease, *Marburgvirus marburgvirus*—composed of Marburg virus (MARV) and Ravn virus (RAVV). Differing by 21% at the nucleotide level [1], these virus strains are highly conserved at the amino acid level except in the glycoprotein gene [2]. By way of contrast, the genus *Ebolavirus* currently has five species that vary 40–50% at the amino acid level: *Bundibugyo ebolavirus* (BDBV), *Reston ebolavirus* (RESTV), *Sudan ebolavirus* (SUDV), *Taï Forest ebolavirus* (TAFV), and *Zaire ebolavirus* (EBOV). It is often the case that antisera raised against one member of the *Ebolavirus* genus may not necessarily react against the others, and cross-reactive monoclonal antibodies can be rare, especially to the glycoprotein [3,4].

While most of the recent outbreaks in Africa were caused by EBOV, other large outbreaks have been caused by SUDV, followed by BDBV and TAFV, all within the lush central African biome. RESTV circulates in the Philippines, and although it is not known to cause disease in humans, it is highly lethal to nonhuman primates and has been imported to the US several times. Unpredictable re-emergence of filoviruses in new locales and evidence of potential new variants in nonhuman hosts [5,6] have rekindled interest in their global distribution. Within varied nucleotides and resulting amino acid sequences, there is secondary and tertiary information that can reveal conserved surface patches. Preservation of these regions implies a pivotal role in viral replication, and these conformations make ideal targets for diagnostics and therapeutics as, no matter the species or country of emergence, there will likely be sufficient homology for cross-reactivity. Because negative-sense RNA viruses are very error prone, a viral component that is not subject to intensive antibody surveillance is further likely to be well conserved through time and across geographies.

We had previously solved the 3-dimensional structure of the MARV nucleoprotein (NP) C-terminal domain using three different llama single-domain antibodies (sdAbs) acting as crystallization chaperones [7]. Each sdAb primarily engaged a highly conserved hydrophobic basin formed by a trio of alpha helices using typical CDR-centric paratopes that resulted in apical approaches. We recently demonstrated cross-reactivity of these anti-MARV sdAb with Měnglà virus NP, a member of a putative new filovirus genus discovered in China [6], and used modeling to reveal that the partially conserved epitope was also likely to be basin-like [8]. Dali homology searching had previously located a similar secondary structure within the *Ebolavirus* genus NP, two of the three alpha helices being identified as forming a dorsal “V”-like shelf and a much smaller, shallower basin, well upstream of the C-terminus [7]. Here, we characterize a trio of semisynthetic anti-*Ebolavirus* sdAb previously selected on live virus preparations and known to recognize the C-terminal region of NP [9] to define the determinants of cross-reactivity and specificity. Our curiosity on how these sdAbs engage NPs was particularly piqued by the absence of an obvious classical deep concave epitope on the dorsal surface, known to be much favored by sdAbs. We first transitioned from live virus to recombinant protein antigens to reconfirm specificity profiles and identify suitable strategies for bait-prey generation of sdAe–NP fragment complexes. X-ray diffraction was then used to solve crystal structures of sdAb–antigen complexes and unbound sdAbs. Molecular modeling, contact mapping, and analysis of ebolavirus NP sequences available in GenBank allowed us to rationalize the observed antigen specificity profiles, define the routes of engagement, and reveal any restructuring required for fit.

## Results

### Antiviral sdAb specificities reside in recombinant NP and NP C-terminal domains

**Fig. 1.**
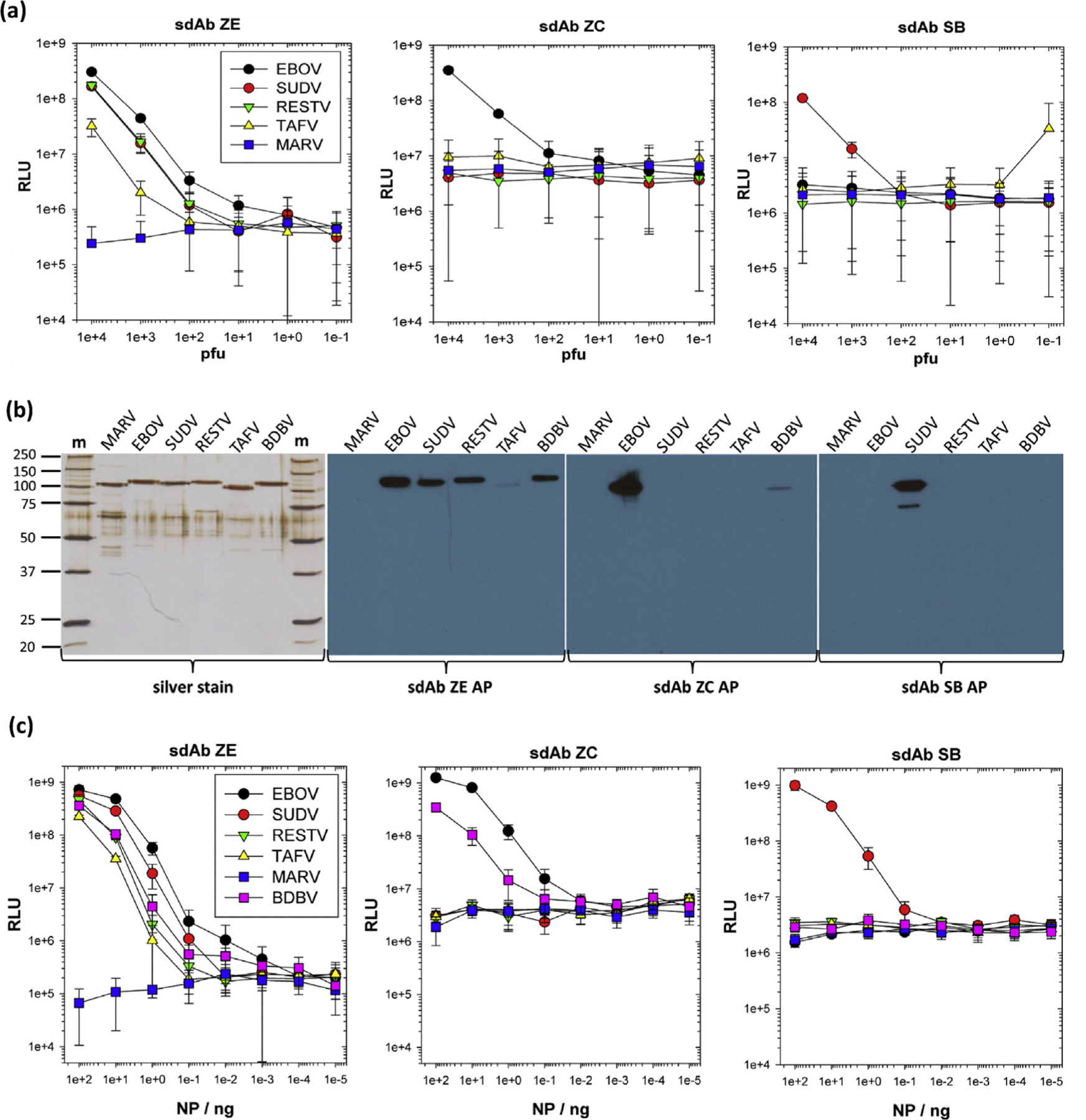
Transitioning from live virus to recombinant NP for reassessment of sdAb specificities. (a) Monoclonal affinity reagent sandwich assay (MARSA) using each of the sdAb as neutravidin oriented captor and phage displayed tracer to reconfirm cross-reactivity profiles on EBOV, SUDV, RESTV, and TAFV and the negative control MARV. The legend is within the sdAb ZE graph and is the same for all panels. The experiment was repeated on two different occasions, and the error bars represent ±SD. (b) Analysis of 250 ng of purified recombinant NP from HEK293T lysates after density gradient centrifugation by SDS-PAGE and silver staining plus Western blotting and probing with 100 nM alkaline phosphatase (AP) fusions of sdAb ZE, ZC, or SB. (c) Titration of recombinant NP within the MARSA using the oriented sdAb captor and phage tracer. The legend is within the sdAb ZE graph and is the same for all panels. The experiment was repeated on two different occasions and the error bars represent ±SD. BDBV, *Bundibugyo ebolavirus*; EBOV, *Zaire ebolavirus*; m, molecular weight marker with sizes in kDa; MARV, Marburg virus; NP, nucleoprotein; RESTV, *Reston ebolavirus*; RLU, relative light units; SD, standard deviation; SUDV, *Sudan ebolavirus*; TAFV, *Taï Forest ebolavirus*;sdAb, single-domain antibody.

We previously established sdAb antigenic specificities using monoclonal affinity reagent assay (MARSA; where the same sdAb is used as passively immobilized captor and phage-displayed tracer) titrations of live virus and crude recombinant NP lysates from HEK293T cells [9]. We had also used Western blotting of virus preparations and recombinant NP within lysates of both *Escherichia coli* and HEK293T cells using dimeric alkaline phosphatase fusions of sdAbs. To be more quantitative, we first repeated the MARSA with the neutravidin-captured biotinylated sdAb to reconfirm the differential reactivity of the three sdAbs on live virus preparations. sdAb genes were first mobilized to pecan126 and *E. coli* HBV88, a combination that conveniently enables both high-level production of singly biotinylated sdAb protein and phage-displayed sdAb by altering arabinose and isopropyl-β-D-thiogalactoside (IPTG) levels [10]. As before, the sdAb ZE demonstrated a broad yet differential cross-reactivity among the *Ebolavirus* genus while the sdAb ZC and SB are more specific for the viruses upon which they were originally selected (Fig. 1a).

We transitioned from live virus to recombinant NP, which enabled us to work at BSL-2 and also allowed us to analyze reactivity against BDBV NP, a virus we did not have to hand. Human codon–optimized NP genes were overexpressed in HEK293T cells and recombinant protein purified from cell lysates by density gradient centrifugation, as described previously [8]. Purity was established by sodium dodecyl sulfate-polyacrylamide gel electrophoresis (SDS-PAGE) and silver staining, while Western blotting using hyperactive alkaline phosphatase fusion proteins of sdAbs ZE, ZC, and SB revealed retention of the differential cross-reactivity of the sdAb ZE and the high specificities of sdAbs ZC and SB (Fig. 1b). The MARSA was repeated using the oriented biotinylated sdAb and phage-displayed sdAb as the tracer (Fig. 1c) showing preservation of the patterns of the binding live virus, confirming the binding profiles seen by Western blotting, and confirmed a small degree of cross-reactivity toward BDBV NP by the sdAb ZC.

**Fig. 2.**
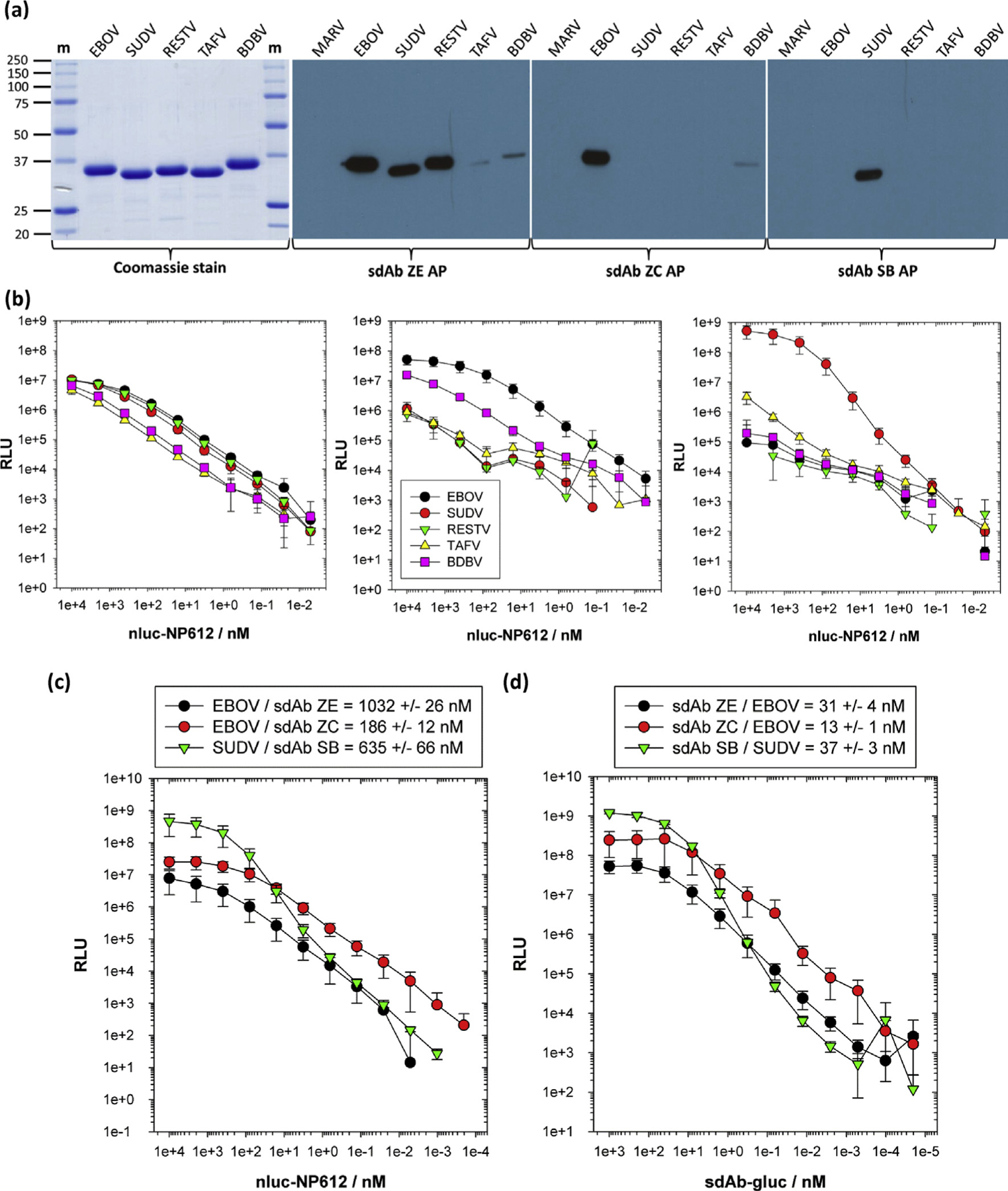
Transitioning to recombinant NP C-terminal fragment binding studies. (a) Coomassie-stained SDS-PAGE of the nluc-NP612 fusion proteins with Western blotting using the sdAb-AP fusions. (b) Titrations of the nluc-NP612 fusion proteins over each of the oriented sdAb as captors. The legend is shown in the sdAb ZC graph and is the same for all panels. ELISA was performed on two different occasions, and error bars represent ±SD. (c) Determining EC_50_ values for the interaction of nluc-NP612 fusions with neutravidin-immobilized sdAb. (d) Determining EC_50_ values for the interaction of sdAb–gluc fusions with passively immobilized full-length NP polymers. ELISAs for (c) and (d) were performed on three different occasions, and error bars represent ±SD, with the EC_50_ values shown in the legend boxes. ELISA, enzyme-linked immunosorbent assay; m, molecular weight marker with sizes in kDa; NP, nucleoprotein; SD, standard deviation; BDBV, *Bundibugyo ebolavirus*; EBOV, *Zaire ebolavirus*; RESTV, *Reston ebolavirus*; SUDV, *Sudan ebolavirus*; TAFV, *Taï Forest ebolavirus*;sdAb, single-domain antibody; nluc, nanoluciferase.

We then assessed the feasibility of generating complexes between the sdAb and NP epitope by generating monomeric nanoluciferase (nluc) fusions of the NP C-termini for Western blotting by sdAb–alkaline phosphatase fusions (Fig. 2a) and capturing using the oriented biotinylated sdAb (Fig. 2b) to determine if the trends of specificity were preserved. The data tended to mirror the virus and recombinant NP capture profiles and Western blot profiles in that the sdAb ZE still possessed broad yet differential reactivity among the NP species. The sdAb ZC was still more specific to EBOV with partial cross-reactivity to BDBV preserved, while SB retains high specificity to SUDV with around three logs reduced signal relative to the TAFV C-terminus. We then titrated the NP612 C-termini as nluc fusions over neutravidin-oriented cognate sdAb to reveal EC_50_ in hundreds of nanomolar (Fig. 2c). Titrating sdAb–gluc fusions (glucibodies) over passively immobilized cognate NP revealed EC_50_ in the tens of nanomolar (Fig. 2d), which is respectable for antibodies from a single-pot library. The approximate 33-, 14-, and 17-fold drop in EC_50_ for sdAbs ZE, ZC, and SB, respectively, when transitioning from full-length NP polymers to C-terminal fragments may reflect the complex antigenic landscape of the passively immobilized virus preparations originally used to select the sdAb and/or the high epitope density favoring rebinding during enzyme-linked immunosorbent assay (ELISA). The tendency of the sdAb SB curves to be more sigmoidal with steeper Hill slopes than sdAbs ZE and ZC throughout, indicating a degree of cooperativity in binding for sdAb SB. Despite the decreases in EC_50_ when transitioning from NP to C-termini, the specificity profiles were essentially preserved for each of the sdAb, indicating sufficient architecture was present to pursue informative antibody–antigen complexes and crystallization trials.

### Generating complexes of sdAb and NP C-termini, free sdAb, and their crystallization: evidence of sdAb SB driving a 2:2 tetrameric antibody–antigen assembly

We coexpressed the His_6_ tag–deficient sdAb with KIHis_6_-tagged NP C-termini in the *E. coli* periplasm to form antibody–antigen complexes *in vivo* and also used periplasmic expression to produce the His_6_-tagged sdAb alone to obtain the unbound forms of the antibodies. The periplasm has only a small proportion of host proteins when compared with the cytosol, offering an immediate enrichment advantage. Immobilized metal affinity chromatography (IMAC), followed by size-exclusion chromatography (SEC), was then used to generate multi-milligram amounts of highly pure complexes from 500-mL shake-flask cultures. The strategy was successful in generating protein preparations that yielded crystals that diffracted for the sdAb ZC with the EBOV NP C-terminus (NP amino acids 634 – end, “EBOV NP634”) and sdAb SB with both the parental SUDV NP C-terminus (NP amino acids 610 – end; “SUDV NP610”) and also a trimmed-down version (NP amino acids 634 – end; “SUDV NP634”) that reduced regions exhibiting missing electron density. While the sdAb ZE complex with the EBOV NP C-terminus was straightforward to be produced, we failed to generate any crystals using this material. The unbound sdAb ZE was poorly soluble and could only be maintained at high enough concentrations in solution by inserting the 22-aminoacid synuclein tag (syn119–140) [11] between FR4 and the His_6_ tag to make sdAb ZE-syn^119–140^. Despite yield improvements, we were unable to generate a crystal of sdAb ZE-syn^119–140^ alone or after fusion to downstream APEX2, maltose-binding protein, and even an upstream fusion to maltose-binding protein. However, using a His_6_ tagless version of sdAb ZE-syn^119–140^, we revisited the complex again, and although parental (NP610) and trimmed (NP634) versions of EBOZ NP C-terminus were highly productive in terms of complex yield, we could only obtain crystals with SUDV NP634.

**Fig. 3.**
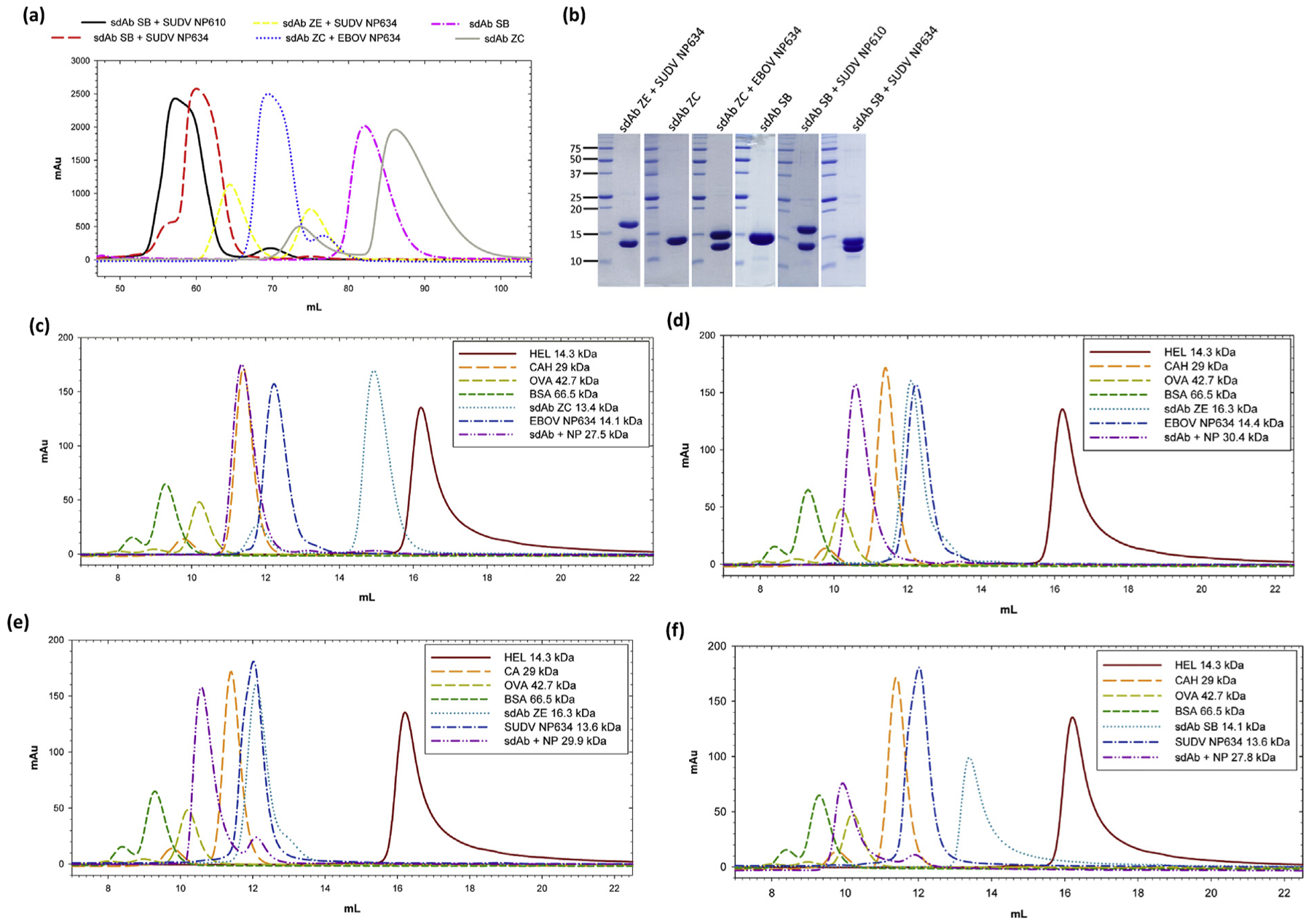
(a) Preparative SEC chromatograms of the final sdAb and sdAb + NP C-terminal complexes used for crystallization trials. (b) Coomassie Blue–stained SDS-PAGE analysis of 20 μg of the final concentrated protein preparations (except sdAb ZC, which was 10 μg). Molecular weight markers (kDa) are indicated on the left. (c)–(f) Overlays of various small-scale analytical SEC of various sdAb, NP634, and sdb + NP634 combinations relative to molecular weight markers; hen egg lysozyme (HEL, 14.3 kDa); carbonic anhydrase (CAH, 29 kDa); ovalbumin (OVA, 42.7 kDa); bovine serum albumin (BSA, 66.5 kDa). (c) sdAb ZC, EBOV NP634, and sdAb ZC + EBOV NP634. (d) sdAb ZE, EBOV NP634, and sdAb ZE + EBOV NP634. (e) sdAb ZE, SUDV NP634, and sdAb ZE + SUDV NP634. (f) sdAb SB, SUDV NP634, and sdAb SB + SUDV NP634. SEC, size-exclusion chromatography; EBOV, *Zaire ebolavirus*; SUDV, *Sudan ebolavirus*; sdAb, single-domain antibody.

The preparative SEC curves of the material that yielded crystals in this study are shown in Fig. 3a, with SDS-PAGE analysis in Fig. 3b. The noticeable shifts in the elution profiles for the sdAb–SB–NP complexes were rationalized by analytical SEC, where we purified individual components separately (these sdAbs were all His_6_ tagged), combined in the 1:1 M ratio or left individually for 1 h, and then injected into the column. Fig. 3c shows a model profile of globular sdAb ZC (13.4 kDa) migrating toward the size of hen egg lysozyme (HEL; 14.3 kDa), whereas the extended conformation of the EBOV NP634 (14.1 kDa) results in faster-than-predicted elution, although the final complex (27.5 kDa) migrates with carbonic anhydrase (CAH; 29 kDa). If we substitute the sdAb ZC for ZE (Fig. 3d), a protein we could not crystallize and thus may have considerable disorder, we see the sdAb and EBOV NP634 each eluting faster, with the complex (30.4 kDa) consequently eluting between ovalbumin (OVA; 45 kDa) and CAH (29 kDa). Now substituting the EBOV NP634 for the SUDV NP634, we see essentially the same profile (Fig. 3e), indicating the characteristics of the complex are not perturbed by the change in NP species. We observe a small amount of unbound SUDV NP634 at the 12-mL mark, indicating the lower affinity that the sdAb ZE has for this species over the cognate EBOV NP634. Finally, substituting the sdAb ZE for the sdAb SB, partially disordered because we have 5 amino acids missing electron density in CDR1 for the unbound form, we see a more conventional elution profile for the sdAb in between the sdAb ZE and sdAb ZC (Fig. 3f), which should result in the complex with SUDV NP634 migrating analogous to either of the sdAb ZE complexes if not slightly slower. However, the peak elutes well beyond OVA (42.7 kDa) toward bovine serum albumin (BSA; 66.5 kDa), suggesting that the sdAb SB may be driving the formation of a multimeric SUDV NP634 complex. Because the 1:1 complex would be expected to be 27.8 kDa, a dimeric 2:2 complex at 55.6 kDa is not inconceivable at this elution point. Statistics of the crystal structures are shown in Table 1.

### Atypical approaches are used by all three sdAbs to engage the NP C-terminus

**Fig. 4.**
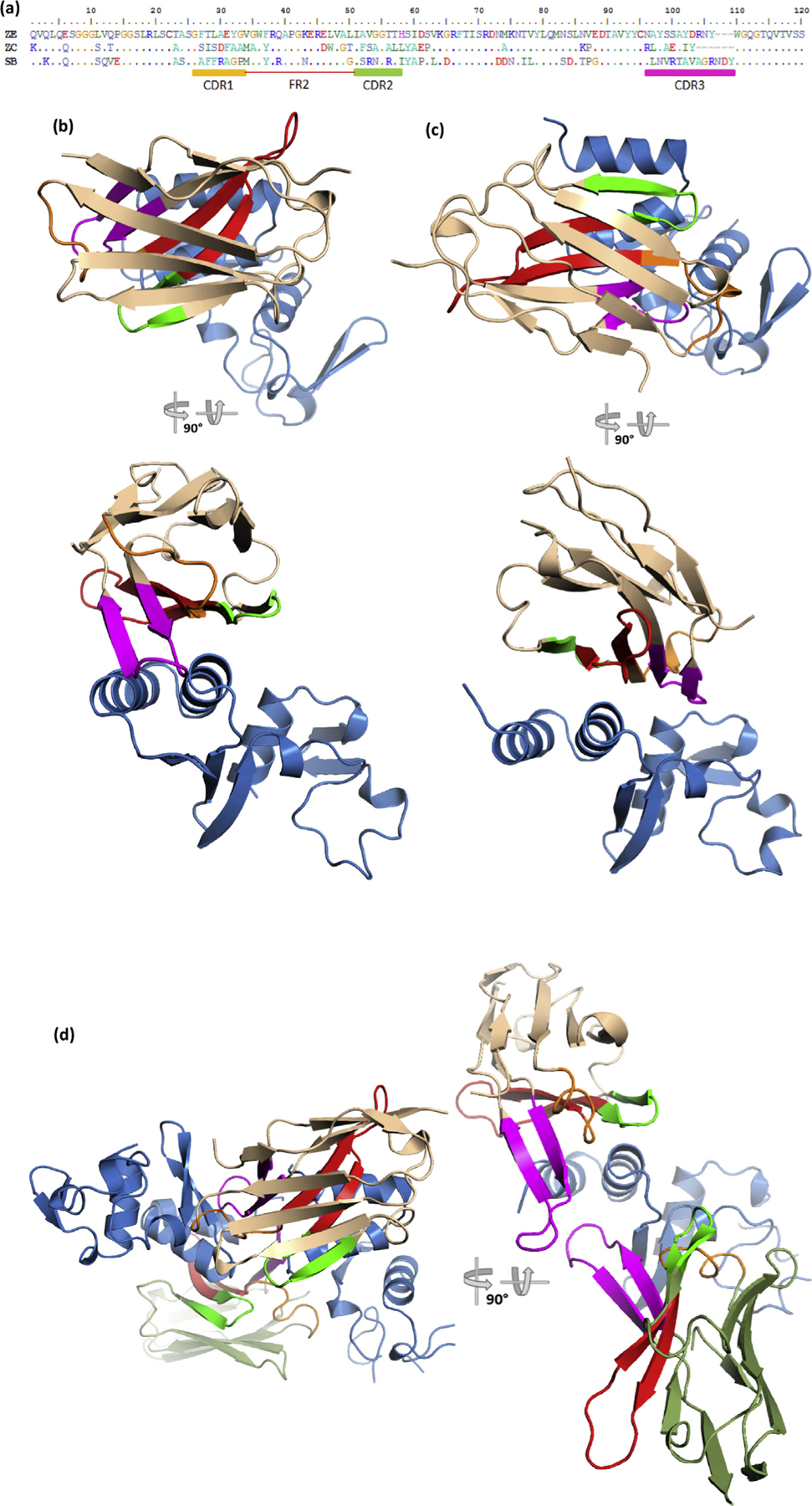
(a) Amino acid sequences of the three sdAbs under study with CDR and FR boundaries denoted and colored; CDR1, orange; CDR2, green; CDR3, magenta; FR2, red. “Top down” and “side-on” views of the antibody–antigen complexes of (b) sdAb ZE + SUDV NP634, (c) sdAb ZC + EBOZ NP634, and (d) sdAb SB + SUDV NP634. “Side-on” view of the sdAb SB complex has one of the NP634 components removed for clarity to show the long CDR3 loops of both sdAbs more clearly. Colors denoted are as follows; sdAb, wheat; NP634, sky blue; for the sdAb SB complex, the alternative sdAb is colored moss green; CDR and FR colors matches the primary structure. EBOV, *Zaire ebolavirus*; SUDV, *Sudan ebolavirus*; sdAb, single-domain antibody; FR2, framework 2.

The amino acid sequences and CDR/FR boundaries of the sdAb are shown in Fig. 4a and color coordinated with the structural representations. The overall approaches used by the sdAb to bind the NP C-terminus are shown in Fig. 4b–d. sdAbs ZE (b) and ZC (c) reveal an atypical “side-on” approach to antigen, with each sdAb using framework 2 (FR2) scaffolding at the interface. Each sdAb engages the NP C-terminus from diametrically opposed directions. The sdAb ZE deploys its CDRs toward the N-terminus of the NP domain to overlay the V-shelf very thoroughly. In contrast, the sdAb ZC deploys its CDRs toward the C-terminus, reaching downstream of the V-shelf and shifting its bulk partially from the conserved region. The approach used by the sdAb SB appears more complex in using a 2:2 stoichiometry (Fig. 4d). Structures derived from crystals generated using either SUDV NP610 or SUDV NP634 complexed with the sdAb SB reveal superimposable coordinates in two lattices, suggesting this is not a crystal-packing artifact and mirror the SEC data of a tetramer. Furthermore, the 2:2 sdAb SB + SUDV NP complexes superimpose with a root-mean-square deviation of 1.6 Å over 406 alpha carbon positions while the crystal-packing patterns of the 2:2 complexes (asymmetric units in each crystal) differ between the two crystal forms.

### High conservation of the ebolavirus NP C-terminal domain through history and geography

**Fig. 5.**
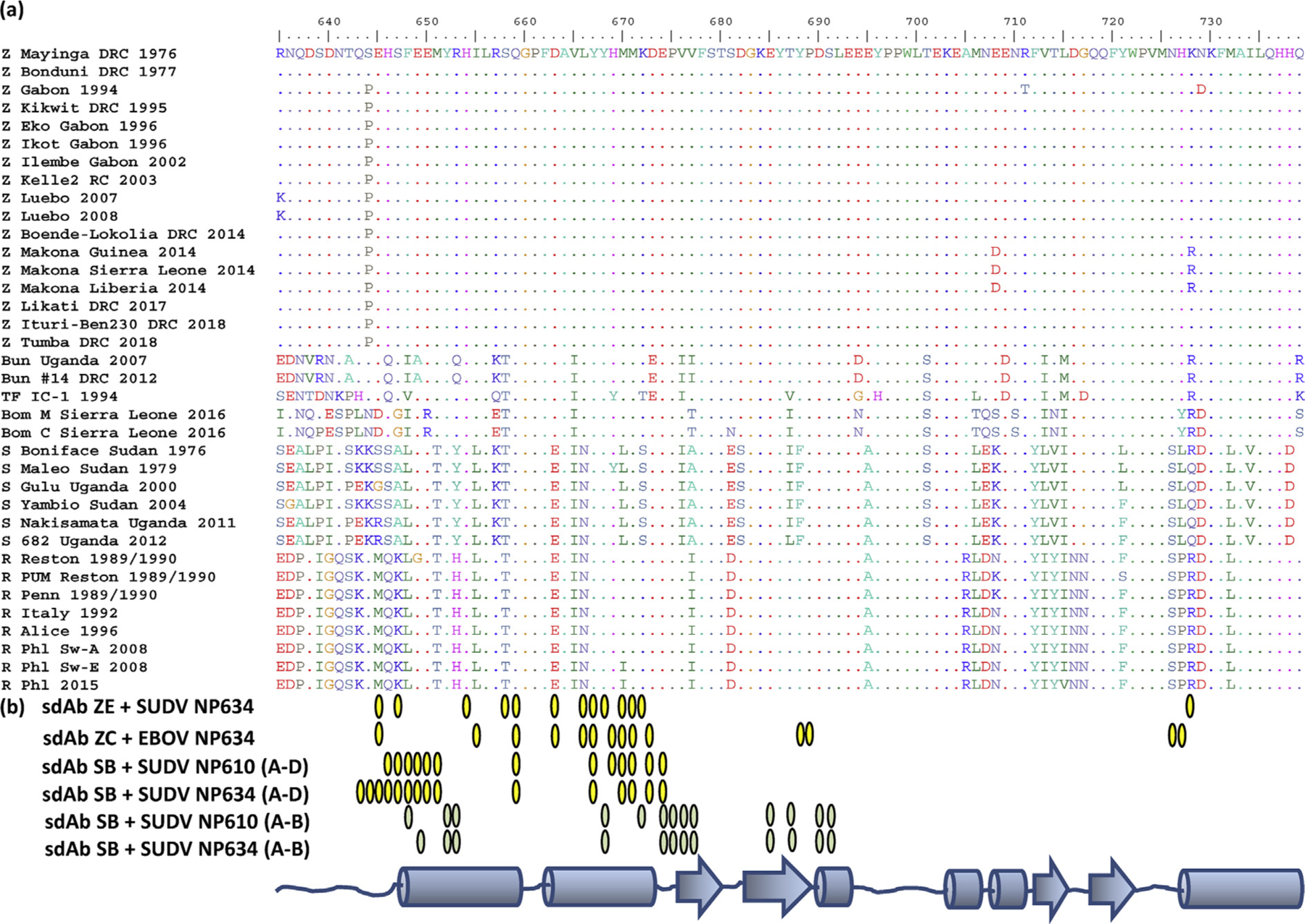
(a) Alignment of the NP C-termini from Ebola virus genomes available in GenBank grouped according to species and then the chronological order. The key to abbreviations of viruses is as follows: Z, EBOV; S, SUDV; Bun, BDBV; TF, TAFV; Bom, Bombali; R, RESTV. Genbank accession number of the sequences used: Z Mayinga DRC 1976, JQ352763.1; Z Bonduni DRC 1977, KC242791.1; Z Gabon 1994, Y09358.1; Z Kikwit DRC 1995, AF054908.1; Z Eko Gabon 1996, KC242793.1; Z Ikot Gabon 1996, KC242798.1; Z Ilembe Gabon 2002, KC242800.1; Z Kelle2 RC 2003, KF113529.1; Z Luebo DRC 2007, HQ613403; Z Luebo DRC 2008, HQ613402; Z Boende-Lokolia DRC 2014, KM519951.1; Z Makona, Guinea 2014, KJ660346.2; Z Makona Sierra Leone 2014, KM034550; Z Makona Liberia 2014, KP178538; Z Likati DRC 2017, MH481611; Z Ituri-Ben230 DRC 2018, MK163663.1; Z Tumba DRC 2018, MH733478; Bun Uganda 2007, NC_014373.1; Bun #14 DRC 2012, KC545396.1; TF IC-1 1994, NC_014372; Bom M Sierra Leone 2016, NC_039345; Bom C Sierra Leone 2016, MF319186; S Boniface Sudan 1976, AF173836.1; S Maleo Sudan 1979, KC242783.2; S Gulu Uganda 2000, Y729654.1; S Yambio Sudan 2004, EU338380.1; S Nakisamata Uganda 2011, JN638998.1; S 682 Uganda 2012, KC545392.1; R Reston 1989/1990, AB050936.1; R PUM Reston 1989/1990, #AY769362; R Penn 1989/1990, AF522874; R Italy 1992, KY798007.1; R Alice 1996, JX477166.1; R Phl Sw-A 2008, FJ621583.1; R Phl Sw-E 2008, FJ621585.1; R Phl 2015, MF540570.1. (b) Schematic of the NP C-terminus secondary structure with interface residues from each of the complexes shown as yellow ovals except for the sdAb SB chains A–B interface colored moss green. The 2:2 tetramers of sdAb SB + SUDV NP610 and sdAb SB + SUDV NP634 each have four interfaces of two distinct types; chain A is one of the sdAbs, while chains D and B are two different NPs. NP, nucleoprotein; sdAb, single-domain antibody; EBOV, *Zaire ebolavirus*; SUDV, *Sudan ebolavirus*; BDBV, *Bundibugyo ebolavirus*; TAFV, *Taï Forest ebolavirus*;RESTV, *Reston ebolavirus*.

To rationalize sdAb specificity, we aligned predicted amino acid sequences of the ebolavirus NP C-terminus from residue 634 until the C-terminus that are available in GenBank from the initial 1976 outbreaks to the present day (Fig. 5), also leveraging recent works to select representative sequences [12–14]. This stretch of NP was the major fragment used herein, and we only visualize electron density from residues 637 onward for the sdAb ZE + SUDV NP634 complex and residues 643–645 onward for the other complexes. Within each species, this region of NP appears highly conserved among isolates along the whole length, with both EBOV and SUDV showing very little variation in over 4 decades since their first documented emergence. Mutations such as E708D and K728R have tended to be conservative occurring during the Makona 2014 outbreak, although S644P of 1994 and subsequent EBOV isolates appear nonconservative yet lies just outside our region of interest. Between species, there is generally a trend toward conservation, particularly the more buried residues that would be responsible for maintaining the overall architecture of the region.

### sdAb ZE SUDV NP634: differential ebolavirus specificity explained

**Fig. 6.**
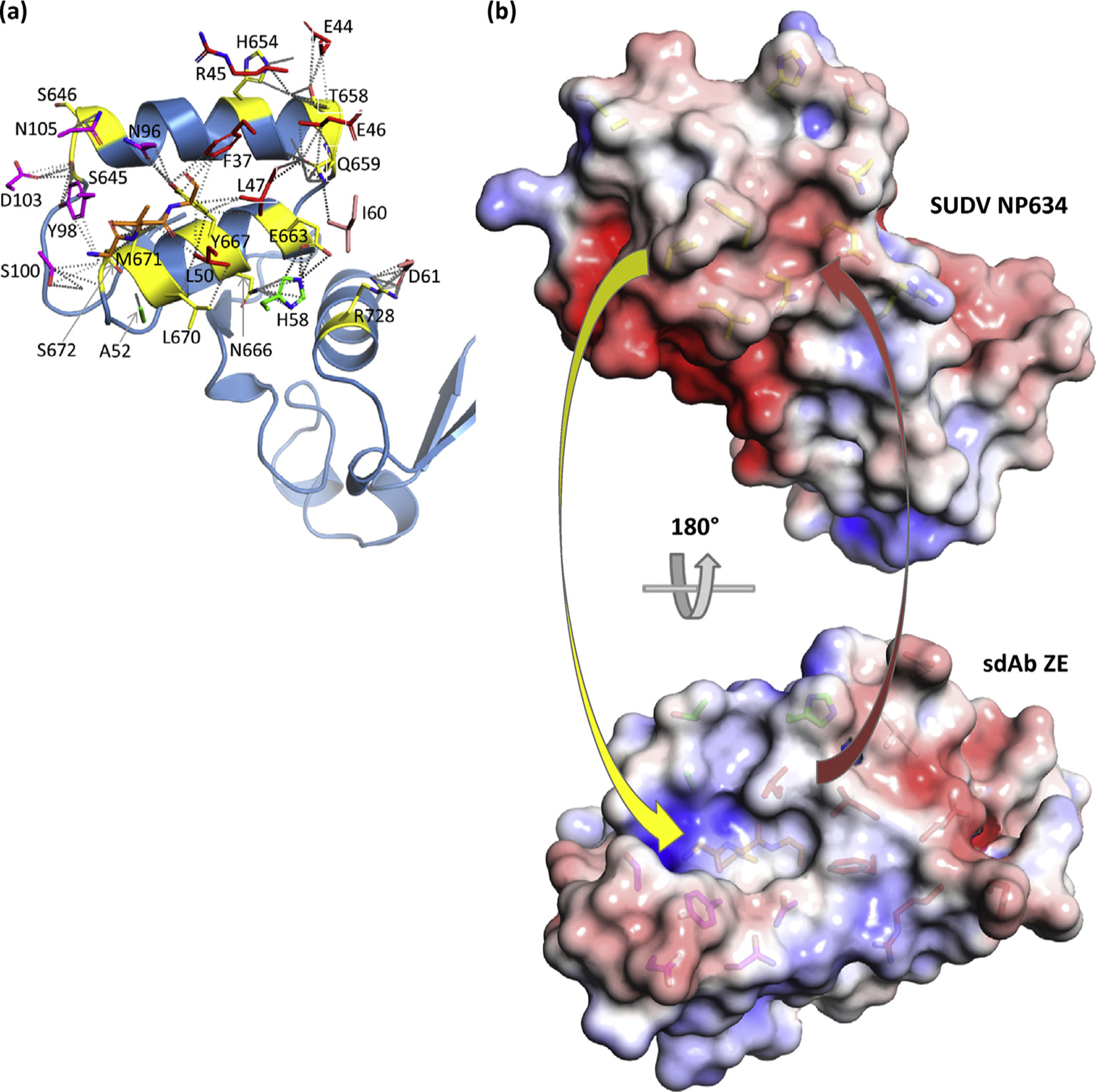
Engagement of SUDV NP634 by sdAb ZE. (a) NP634 is shown in sky blue color with interface residues colored yellow, while sdAb interface residues are colored as follows: CDR1, orange; CDR2, green; CDR3, magenta; FR2, red; and FR3, salmon. For the GVG stretch in CDR1, the main chain is shown although is not labeled for clarity. (b) Electrostatic surfaces with the scale ranging from −5 (red) to +5 (blue) K_b_T/e_c_). Top: antibody’s eye view of the epitope on SUDV NP634. Bottom: NP’s eye view of the sdAb paratope. Arrows indicate some of the major complementarities that are visible: yellow, Y667 and M671 to the sdAb recess; red, FR2 L50 and L47 to the NP shallow basin. SUDV, *Sudan ebolavirus*; sdAb, single-domain antibody; NP, nucleoprotein; FR2, framework 2.

To look more closely at the relative contributions of CDR and FR to binding, Arpeggio [15] was first used to display all interactions within 4 Å between interface residues identified by PDBSum [16]. Fig. 6a reveals the molecular basis for the cross-reactivity of the sdAb ZE with Q659, Y667, and M671, spanning the length of the V-shelf and which are conserved in all ebolavirus species, serving as major interface residues. Q659 hydrogen bonds and has nonbonded contacts with L47 plus nonbonded contacts to E46 and I60 (of FR3); centrally located Y667 hydrogen bonds and has nonbonded contacts with N96 plus nonbonded contacts with F37, L47, and L50; M671 has multiple nonbonded contacts with G33, V34, and G35 of CDR1, Y98, and S100 of CDR3. Less conserved, yet major, interface residues are E666 and R728; E663 salt bridges, hydrogen bonds and has nonbonded contacts with H58 of CDR2 and occurs in RESTV and SUDV NP although in other species, the position 663 is D, still preserving the carboxyl R-group; similarly, R728 salt bridges, hydrogen bonds, and has nonbonded contacts with D61 of FR3 and is conserved in TAFV, BDBV, and EBOV Makona (but is K in other EBOV isolates), SUDV Boniface (but is Q in other SUDV isolates). Fig. 6b shows the resulting surface topography for the sdAb ZE with a large recess or gully formed by a sunken CDR1 adjacent to CDR3, CDR2, and FR2 residues accommodating the protruding Y667 and M671 of NP while the other face of CDR3 engages the wider end of the V-shelf. Although FR2 L47 and L50 occupy the shallow basin on NP, the overall disposition of the epitope is not especially concave, with the sdAb tending to straddle the varied topographies using a broad mix of CDR and FR engagement.

### sdAb ZC EBOV NP634: high specificity with minimal restructuring

**Fig. 7.**
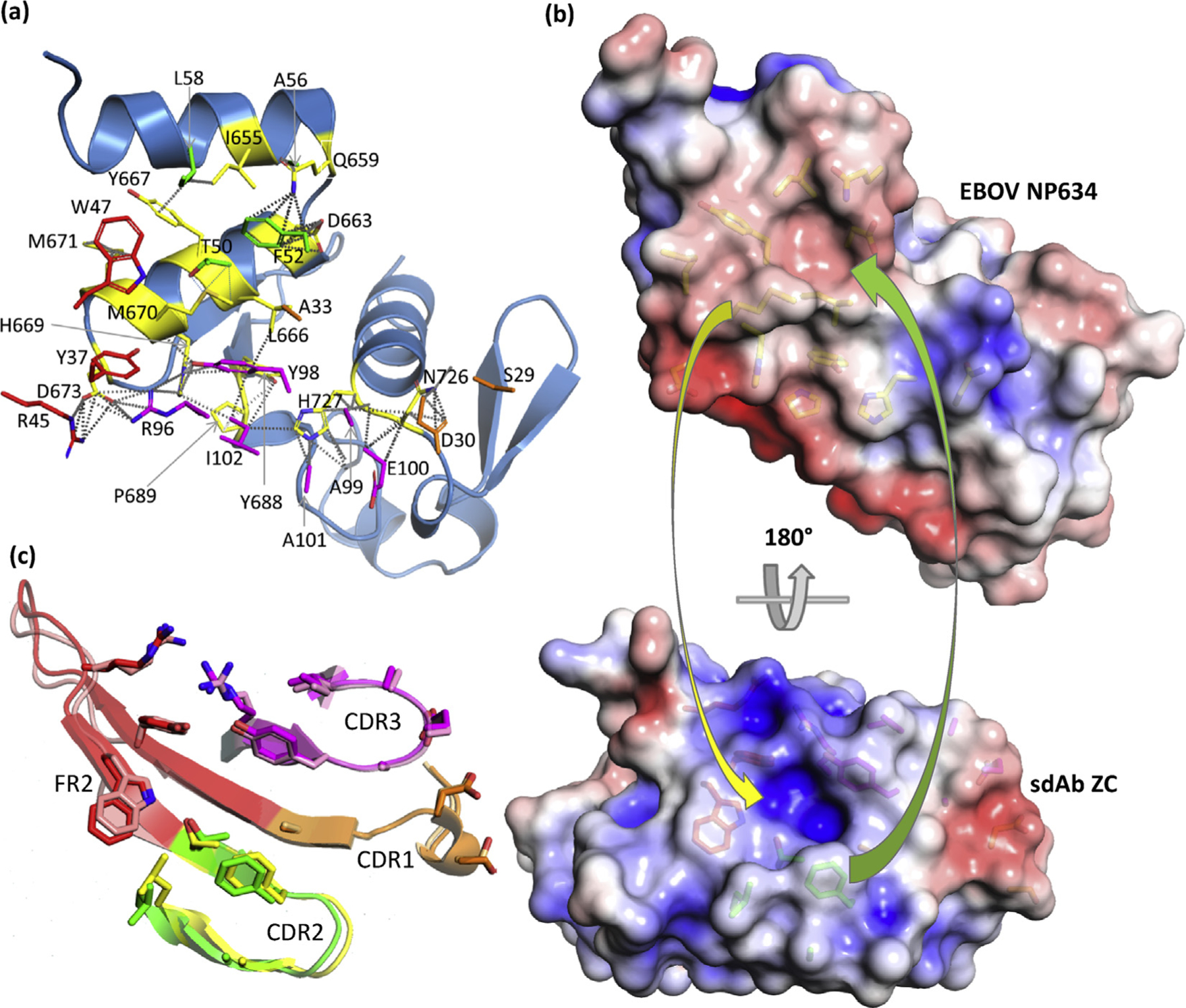
Engagement of EBOV NP634 by sdAb ZC. (a) NP634 is shown in sky blue color with interface residues colored yellow, while sdAb interface residues are colored as follows: CDR1, orange; CDR2, green; CDR3, magenta; FR2, red; and FR3, salmon. (b) Top: antibody’s eye view of the epitope on EBOV NP634. Bottom: NP’s eye view of the sdAb paratope. Arrows indicate some of the major complementarities that are visible: yellow, M670 to the sdAb recess; green, CDR2 F52 to the NP basin. (c) Overlay of the sdAb ZC CDRs and FR2, aligned with the NP634’s eye view as cartoons with the bound/unbound color scheme as follows; CDR1, orange/beige; CDR2, green/yellow; CDR3, magenta/pink; FR2, red/salmon. EBOV, *Zaire ebolavirus*; sdAb, single-domain antibody; ZC, NP, nucleoprotein; FR2, framework 2.

For the sdAb ZC (Fig. 7a), the antibody footprint is shifted from the V-shelf and decentralizes access to Y667 and M671 to interface with residues that are less conserved, leaving less consistent anchor points across the *Ebolavirus* genus; while M670 hydrogen bonds to T50 and Y37 and has nonbonded contacts to both residues along with A33 and Y98, M is substituted for L in SUDV and I in some RESTV isolates; D673 is hydrogen bonded, salt-bridged, and has nonbonded contacts with R96 and R45 although it is substituted for the bulkier E in BDBV and TAFV; H727 hydrogen bonds, has nonbonded contacts with A99, and nonbonded contacts with A101 plus E100 and is conserved in EBOV, BDBV, and TAFV; similarly, N726 shares hydrogen bonding with D30 and nonbonded contacts with D30, A99, E100, and S29 and is conserved in EBOV, BDBV, and TAFV. Discrimination between BDBV and TAFV may be rationalized by H669, which hydrogen bonds and has nonbonded contacts to Y98, but is substituted for Y in TAFV (and SUDV Maleo). While there are interface residues that occur in all species, they are fewer and not predicted to be major contributors, explaining the sdAb ZC’s preference to EBOV NP. Fig. 7b further shows trending toward a smaller recess in the sdAb that would be a closer fit for M670 and, conversely, a smaller recess on NP for accommodating F52 that is not supported by a flexible GVG constellation as in the sdAb ZE. The lower EC_50_ of the sdAb ZC can be rationalized by the minimal restructuring evident between bound and unbound interface residues (Fig. 7c).

### sdAb SB SUDV NP634/610 paratope duality: one paratope with two mutually exclusive epitopes

**Fig. 8.**
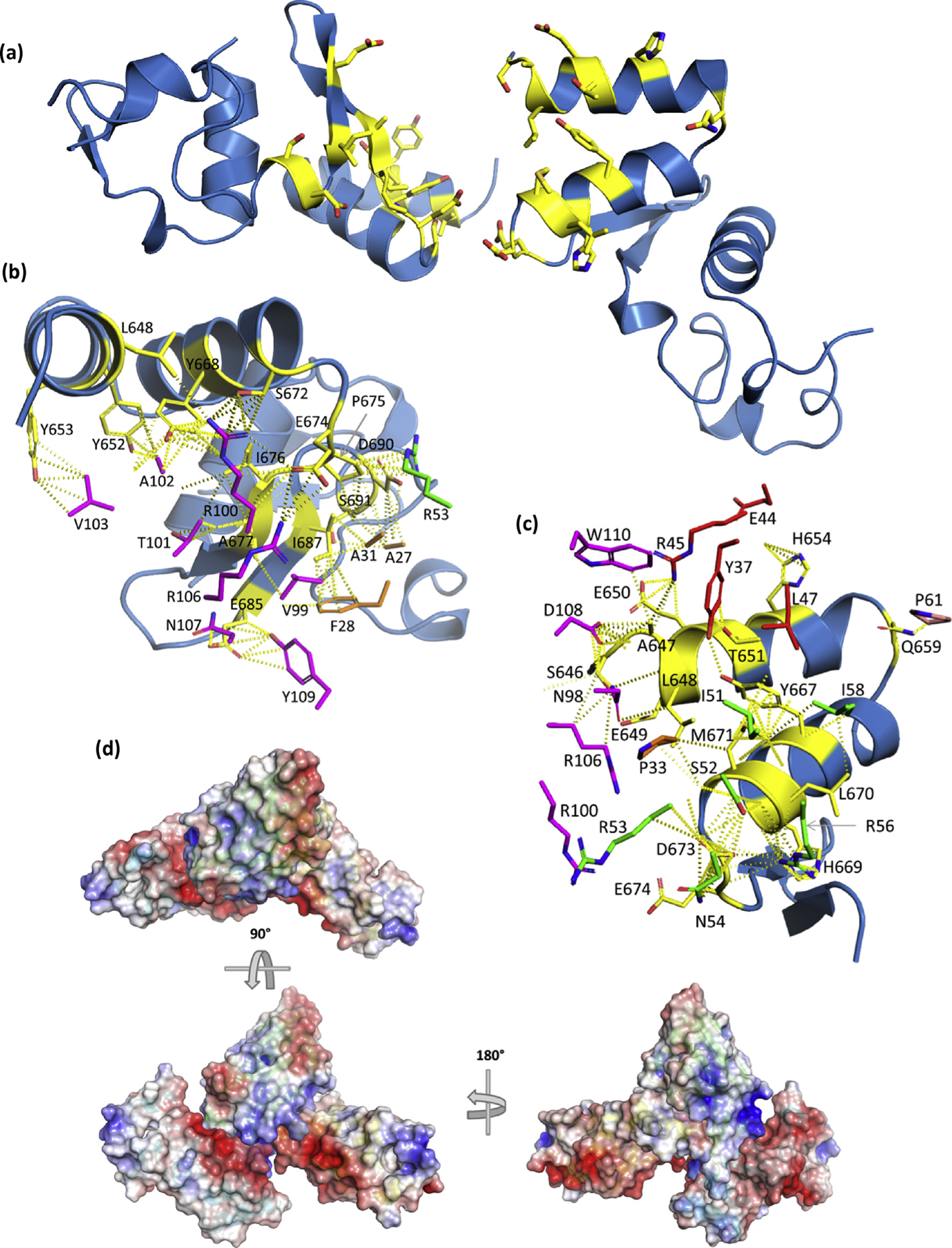
(a) View of the NP C-termini from the sdAb SB + SUDV NP610 complex with the “rightmost” domain (chain D) displayed with the V-shelf uppermost while the “leftmost” domain (chain B) appears almost 180° flipped in a head-over-tail fashion along the axes of the alpha helices of the shelf. Interface residues are colored as yellow sticks. The interface with sdAb (chain A) is shown for chain B (b) and chain D (c) with the partnering NP domain and other sdAb removed for clarity. sdAb interface residues are colored as follows: CDR1, orange; CDR2, green; CDR3, magenta; FR2, red; and other FR, salmon. (d) Electrostatic surfaces of two NP domains with one sdAb (the other removed for clarity) oriented in a “top-down” view aligned as in (a) with sdAb cartoon colored green, while one NP is cyan and the other is yellow. Rotating the assembly 90° and then 180° highlights the arrangement of one NP domain relative tothe other and the long CDR3 loop lodged in the intervening space of the latter. sdAb, single-domain antibody; SUDV, *Sudan ebolavirus*; NP, nucleoprotein; FR2, framework 2.

For the sdAb SB, we have a more complex situation where two distinct modes of binding appear to be used by each sdAb molecule. In each of the SUDV NP610 and NP634 complexes, the approaches used by each of the two sdAbs appear essentially equivalent, and we will focus on chain A for convenience. Fig. 8a reveals how the two domains, with chain D (right) viewed in the same aspect as for sdAbs ZE and ZC, are disposed to each other with the regions bound by one sdAb colored yellow. There are no interfaces identified by PDBSum that occur directly between the NP domains, suggesting the sdAb is eliciting tetrameric complex formation and drawing the NP domains together (as also shown by our SEC data earlier). Between the sdAbs, only R100 and R106 display nonbonded contacts, suggesting this is not a packing artifact or one of the sdAb dimerizations (which we have never observed in any of the 200 sdAbs to date, without resorting to bespoke engineering). Details of each section of the region bound by the sdAb with interfacing residues reveal a more apical CDR3 approach addressing the underside of the V-shelf (Fig. 8b), while the atypical side-on approach with large FR2 involvement occurs on the topside of the V-shelf (Fig. 8c) akin to the sdAb ZE and ZC. Fig. 8d serves to show one of the sdAbs engaging two of the NP domains, with one aspect clearly revealing the protruding CDR3 loop reaching between the domains.

**Fig. 9.**
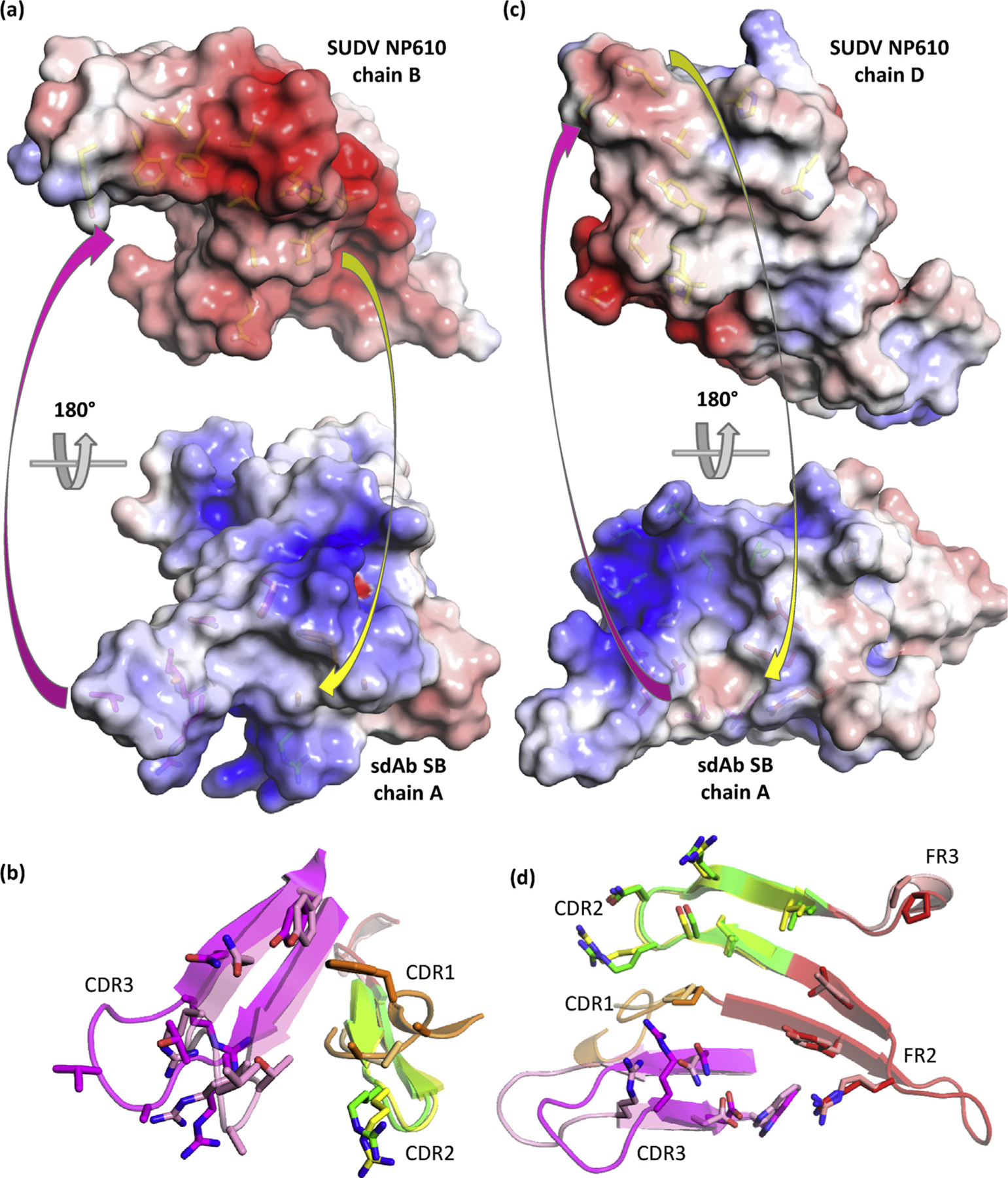
Engagement of SUDV NP610 by sdAb SB. (a) Top: antibody’s eye view of the epitope on chain B of NP610. Bottom: NP’s eye view of the sdAb paratope. Arrows indicate some of the major complementarities that are visible. (b) Overlay of the sdAb SB CDRs and FR2, aligned with the NP634’s eye view as cartoons with bound/unbound color scheme as follows: CDR1, orange/beige; CDR2, green/yellow; CDR3, magenta/pink; and FR2, red/salmon. (c) Top: antibody’s eye view of the epitope on chain D of NP610. Bottom: NP’s eye view of the sdAb paratope. Arrows indicate some of the major complementarities that are visible. (d) Overlay of the sdAb SB CDRs, FR2 and FR3, aligned with the NP634’s eye view as cartoons with bound/unbound color scheme as mentioned previously. SUDV, *Sudan ebolavirus*; NP, nucleoprotein; sdAb, single-domain antibody; FR2, framework 2.

Fig. 9a shows the approach for the sdAb SB interacting with chain B of SUDV NP610, where the CDR3 apex lodges in a hydrophobic cavern underneath the V-shelf with V103, A102, and R100 interfacing with multiple aromatic residues of NP. A substantial amount of restructuring occurs in the CDR3 loop between free and bound sdAbs, with a more compacted unbound loop tending to splay out in a toggle bolt–like manner (Fig. 9b). The second interaction for the sdAb SB is with chain D of the SUDV NP610, where it is more akin to sdAbs ZE and ZC via a side-on aspect over the V-shelf with large contributions from FR2 residues (Fig. 9c). The CDR3 loop spills over the N-terminal region and interfaces with chain D using the opposite face of the CDR3 loop to chain B interfacing, less apical and more toward the take-off and landing sites and leverages some restructuring (Fig. 9d). A large gully is evident in the sdAb that accommodates a ridge that traverses NP610. Similar features are present in the crystal structure of the sdAb SB + SUDV NP634 complexes (Fig. 1a–d, Supplementary Information) with high shape complementarity still evident with the CDR3 apex probing the NP cavern and the sdAb possessing a deep gully. Minor differences are noted in the precise disposition of residues in both the sdAb and NP, alluding to a high degree of plasticity between the antigen and antibody.

The absolute recognition specificity of the sdAb SB is complex to rationalize when we consider the side-on binding mode of chains AD shares common contacts M671, Y667, H654, and others with cross-reactive sdAb ZE. Yet examination of the deep gullying in this paratope shows it would unlikely to be a good fit for the EBOV NP634 of Fig. 7b. Interfacing with residues before F648 is evident in this approach and enters into larger species–specific regions, just upstream of epitopes previously used by others as peptides to generate species-specific antisera in rabbits [17] and monoclonal antibodies derived from mice [18]. The more apical mode of binding between the sdAb SB and chain B is likely to be disturbed by Y653 mutating to R (EBOV and TAFV), Q (BDBV), or H (RESTV), reshaping the cavern into which CDR3 probes. The noticeable collar using A677 may also be deformed by mutating to V (EBOV and TAFV) or I (BDBV and RESTV). If either sdAb has a nonoptimal fit on one of the epitopes, it may be unable to fit the other, diminishing cooperativity and opportunities for binding.

### sdAb approaches to engage NP differ from those used by a Fab

**Fig. 10.**
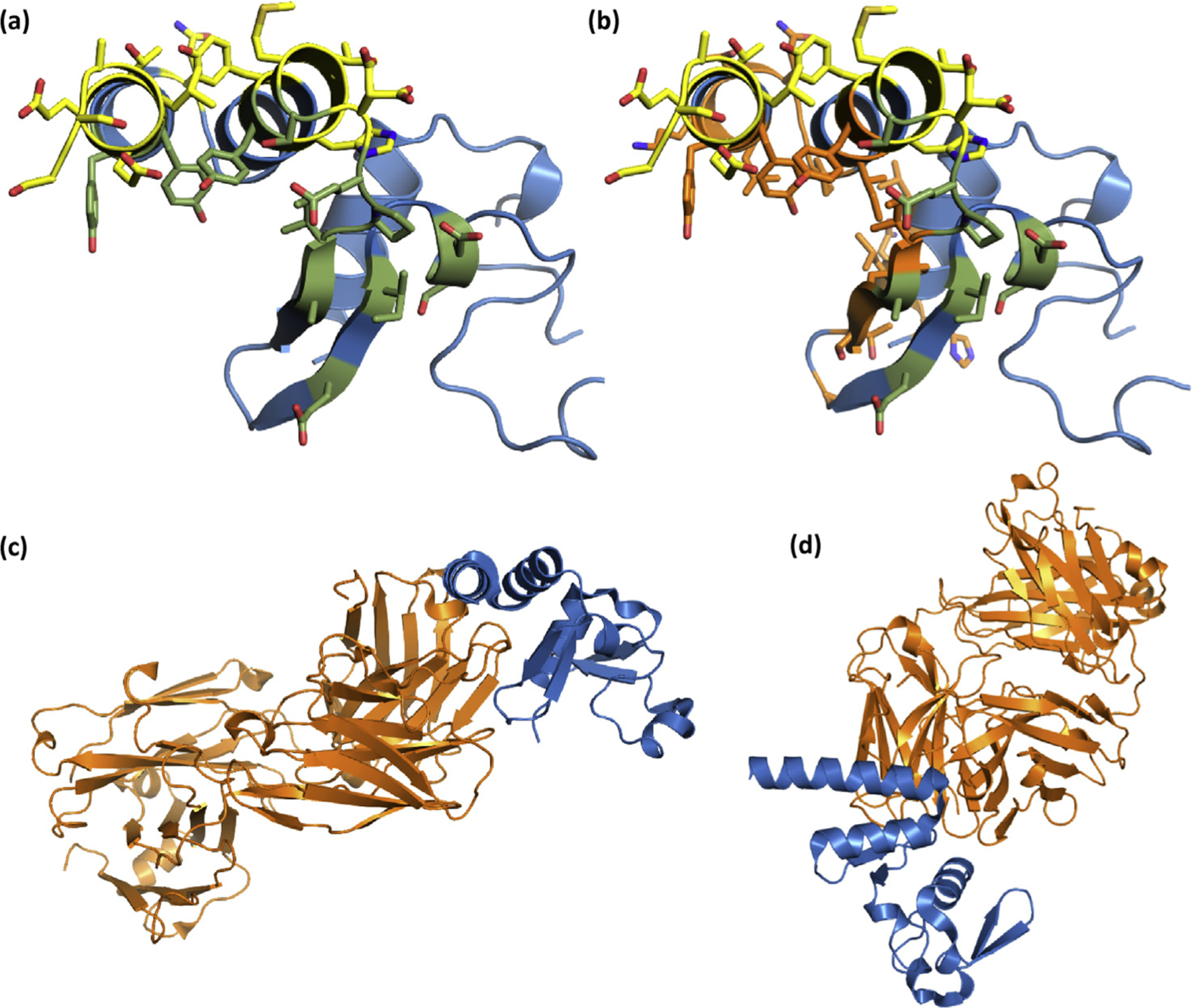
(a) SUDV NP610 with sdAb interfaces colored yellow for the apical approach and moss green for the cavern approach as would occur when 2 sdAbs bind each of 2 NP domains in the tetramer. (b) As for (a), except the interface residue locations predicted by PDBSum to be used by Fab MJ120 are colored orange. (c) The entire Fab (orange) is shown with the NP positioned as in (a) and (b), while (d) represents our typical “top-down” view of the NP V-shelf and entire Fab. SUDV, *Sudan ebolavirus*; NP, nucleoprotein; sdAb, single-domain antibody.

Unlike our MARV work, where no classical Ig-like antibody–NP complex structure existed, the recent elucidation of a synthetic human Fab-binding BDBV NP (PDB 5VKD) [19] enabled us to compare and contrast the approaches used by sdAb and Fab to engage filoviral NP. The synthetic human Fab was isolated by phage display on the recombinant EBOV NP C-terminus to yield a differentially cross-reactive binder in the order of the strongest to weakest as follows: EBOV ≥ TAFV > RESTV > BDBV > SUDV. The ranking contrasts with our differentially cross-reactive sdAb ZE: EBOV > SUDV > RESTV = BDBV > TAFV, indicating different antibody–antigen interactions occur. While no live virus capture data were presented, the Fab was shown to recognize an EBOV minigenome replicon within transfected cells by fluorescence microscopy, suggesting that it is likely to recognize authentic viral NP at least within the context of an infected cell. Fig. 10a is a simplified version of Fig. 8b and c, essentially a combination of our sdAb SB epitopes mapped onto a single NP domain as would occur with both sdAbs engaging in the tetramer, where the cavern interface is colored moss green and the apical interface is colored yellow. We chose the sdAb SB because the V-shelf epitope shares similarities with that of the sdAb ZE yet also provides us with the opportunity to display both atypical and typical epitopes. Fig. 10b shows the interface residues (positions, because SUDV varies from BDBV slightly) for the Fab, as deduced by PDBSum, colored orange, indicating some overlap occurs with the cavern-binding sdAb but appears to be more toward the closed end of the V-shelf. The difference in approach, taken by the Fab to bind NP, is clearly seen in Fig. 10c and d, where the route is off to one side of the V-shelf. An unpublished PDB 5W2B from the same group revealing a Fab binding the RESTV C-terminus shows a very similar approach. Because a Fab is four times the mass of our sdAb and uses a totally different approach, it will be of great interest to determine if it is able to function in virus capture assays to inform us of diagnostic utility and the disposition of the epitope on the ribonucleocapsid.

## Discussion

The puzzle of how an sdAb engages a surface of the ebolavirus NP C-terminus that does not possess the classical deep concave epitope is revealed through our studies of sdAbs ZE and ZC and the nonapical approach of the sdAb SB where several FR residues contribute to shape complementarity and fit. FR involvement has been noted in the earliest of the sdAb specific to an amylase [20] and a hapten [21] and has been commented on recently as a significant contributor to sdAb–antigen interfacing after several large analyses of publically available crystal structures [22–24]. In our present study, we show the consequences of this FR interfacing in recontouring the sdAb surface to be more accommodating to undulating and convex antigenic landscapes, bypassing the need for a concave epitope. The fact that the sdAb SB can use both FR-centric and CDR-centric concave epitope seeking approaches simultaneously indicates these are not necessarily mutually exclusive.

While we cannot rule out concerns that we may not have the complete interaction map based on using a small portion of the NP C-terminus and CDR loop functions may not be completely captured for sdAbs ZE and ZC, our binding studies show that the determinants of specificity relative to virus are retained, and thus, our current maps are still relevant. We were unable to generate a sufficient dynamic range for establishing EC_50_ values by probing ELISA plates coated with the virus (to mimic the original panning process) or generating sufficient recombinant ribonucleocapsids (by coexpression with VP35 and 24 [25]) for glucibody probing, to discover contributions to binding that might be offered by the larger macromolecular structures these sdAbs were selected on. It is tempting to speculate that the sdAb SB, the only clone isolated 24/24 times from phage panning on SUDV thwarted competitors by occupying all the epitope options available and may reflect the native organization of the NP C-terminal domain in the ribonucleocapsid. The compact size of the sdAb lends itself well to exploring surfaces of pathogens not readily available to most IgG, including canyons in poliovirus capsids [26] or in between dense glycoprotein matrices of trypanosomes [27], and the sdAb SB may have extended this capacity to probing macromolecular complexes within pathogens.

The sdAb SB is, to our knowledge, unique in the world of antibodies where a single-domain paratope contains sufficient information to bind two epitopes simultaneously, without overlap, without engineering, and bizarrely does it on the same antigen! While the “two-in one” concept of one antibody binding two epitopes through Fab engineering and directed evolution [28,29] or heterologous IgG chain partnering has been developed into “three-in-one” [30] and even “four-in-one” [31] specificities, these are not trivial undertakings and result in large molecules likely to be costly to produce. Furthermore, the Fab architecture with 6 CDRs offers a larger surface area more likely to accommodate two epitopes without overlap, whereas the minimalist sdAb [32] has half as many CDRs, probably a key factor in their leveraging FR residues in paratope composition. For our immediate needs, the compact architecture of the sdAb lends itself well to facile engineering and inexpensive production in *E. coli* to try and exploit this remarkable capacity for paratope duality in targeting neighboring components within other macromolecular complexes of interest.

## Materials and Methods

### General cloning

Recombinant DNA methods were in accordance with established procedures and used commercially available reagents: Phusion High-Fidelity DNA Polymerase (Thermo Fisher, Waltham, MA); restriction enzymes and β-agarase (New England BioLabs, Beverly, MA); T4 DNA ligase, CIP, and T4 PNK (Roche, Nutley, NJ); GTG low melting temperature agarose for in-gel cloning, (Lonza, Walkersville, MD); oligonucleotides and gBlocks® (Integrated DNA Technologies, Coralville, IA); Assemblies involving polymerase chain reaction (PCR) amplification were sequenced through the inserts and junctions to verify the desired construct. Cloning was carried out in XL1-Blue cells. Full details of cloning, oligonucleotides, maps, and sequences of the resulting constructs are available on request.

### Production and purification of sdAb proteins, glucibodies, and sdAb–NP complexes

Genes encoding sdAbs ZE, ZC, and SB were mobilized to pecan 126 [10] *via Sfi*I *Not*I for production of biotinylated sdAb within *E. coli* HBV88. For production of the unbound sdAb for crystallization, the genes were mobilized to pecan73 [10] (encoding AAA His_6_ after FR4 TVSS). For production of glucibodies, the sdAbs were mobilized to pecan35 [7]. NP C-terminal fragments were either amplified by PCR or obtained as gBlocks® with a KIHis_6_G3S sequence [33] before the region of interest and mobilized *via Nco*I *Hin*dIII to pecan236, a hygromycin-resistant derivative of pecan73 with a dsbA signal sequence in place of *pel*B. The tagless sdAb for NP complex crystallization was derived by PCR amplification into pecan73 via *Nco*I and *Hin*dIII to form pecan219 with FR4 TVSS ending the sdAb; the ZE synuclein His_6_–tagged construct sdAb ZE-syn^119–140^ was created by inserting the sdAb ZE *via Nco*I and *Not*I into pecan202 that had the synuclein 118–140 sequence [11] and His_6_ as an oligonucleotide bridge between the *Not*I and *Hin*dIII sites; similarly, the tagless sdAb ZE was made by insertion into a similarly made pecan268 lacking the His_6_ portion. These constructs were used in *E. coli* Tuner + pRARE with complexes double transformed and selected for ampicillin and hygromycin resistance. AP fusions had been previously generated [9] and stored at −80 °C.

For production, clones were grown in 50 mL of starter cultures of terrific broth plus 2% glucose at 30 °C overnight with ampicillin (200 μgmL^−1^) and chloramphenicol (30 μgmL^−1^) (and 200 μgmL^−1^ hygromycin for complexes) in 250-mL Bellco baffled flasks. The saturated overnight cultures were poured in to 450 mL of glucose-free medium in 2.5-L Bellco baffled flasks without antibiotics and shaken for 3 h at 25 °C. Expression was induced by addition of IPTG to a concentration of 1 mM for 3 h at 25 °C, and the cells were pelleted (Beckman Allegra 6R swing-out rotor), drained, and weighed. The cells were osmotically shocked [34] by resuspension in 14 mL of ice-cold 0.75 M sucrose in 100 mM Tris-HCl pH 7.5, addition of 1.4 mL of 1 mgmL^−1^ HEL (Sigma), followed by dropwise addition of 28 mL of ice-cold 1 mM EDTA pH 8.0 and swirling on ice for 15 min. Two milliliters of 0.5 M MgCl_2_ was added, swirling was continued for 15 min, and the cells were pelleted. Forty-five milliliters of the supernatant (osmotic shockate) was mixed with 5 mL of 10 × IMAC (IMAC buffer: 0.2 M Na_2_HPO_4_, 5 M NaCl, 0.2 M imidazole, 1% Tween-20, pH 7.5), followed by 0.5 mL of High Performance Ni Sepharose (GE Healthcare, Pittsburgh, PA), and the suspension was gently mixed on ice for 1 h. The resin was pelleted at 3000 rpm for 5 min (Beckman Allegra 6R swing-out rotor) and washed twice with 50 mL of 1 × IMAC solution before elution with 2 mL of 0.5 M imidazole in 1 × IMAC buffer, pH 7.4 in Poly-Prep® columns (Bio-Rad, Hercules, CA). Proteins were concentrated in Amicon 10-kDa ultrafiltration devices (Millipore, Billerica, MA) to 200 or 2000 μL for separation by analytical or large-scale SEC, respectively. Analytical preparations were purified on a Superdex 200 Increase 10/300 GL column (GE Healthcare), and 100 μg of the sdAb was combined with 100 μg of the NP C-terminus for 1 h at room temperature before being applied to a Superdex 75 Increase 10/300 GL column operating in phosphate-buffered saline (PBS). For the individual components, 200 μg was incubated at room temperature for 1 h before gel filtration analysis. Gel filtration standards were obtained from GE Healthcare. Our protein preparations were made to 50% glycerol and aliquoted for long-term storage at −80 °C. Large-scale preparations from multiple flasks were applied to a Superdex 75 16/60 column operating in 10 mM Tris-HCl, 150 mM NaCl, pH 7.5 and concentrated for crystallization trials. Proteins were quantified by micro-BCA assay/UV adsorption and analyzed by SDS-PAGE and Coomassie Blue staining for impurities.

### Production of phage-displayed sdAbs

The clones within pecan126 and *E. coli* HBV88 were grown in 2 × YT + 2% (w/v) glucose at 50-mL scale at 37 °C to an OD650 cm^−1^ of approximately 0.5 and infected with M13KO7 at a multiplicity of 20. After leaving stationary for an hour, arabinose was added to 2000 μg/mL and IPTG to 10 μM, and cultures were shaken for 18 h at 30 °C. Cultures were clarified by centrifugation for 20 min at 4 °C (Sorvall RC 6+, F13 FiberLite rotor) and phagemids precipitated by addition of 8 mL of NAP6 (2.5 M NaCl, 20% w/v PEG 6000) and incubated on ice overnight. Centrifugation for 30 min at 4 °C (Beckman Allegra 6R swing-out rotor) was performed to collect the particles that were resuspended in 900 mL of PBS and 900 μL of glycerol for storage at −20 °C until required.

### Viruses

Filoviruses were handled within the BSL-4 laboratory at Texas Biomed, following all applicable CDC Select Agent regulations and local biohazard and safety committee approval. ,sThe viral isolates employed herein were MARV Musoke 1980, EBOV Kikwit 1995, SUDV Boniface 1976, RESTV Reston 1989, and TAFV IC1 1994. Details of virus amplification, purification, and titration have been previously described [9,35].

### Production and gel probing of recombinant NP proteins

Human codon–optimized genes encoding NP from MARV Musoke 1980, EBOV Kikwit 1995, SUDV Boniface 1976, RESTV Reston 1989, TAFV IC1 1994, and BDBV 2007, their expression vectors, and purification have been described previously [7–9,36]. Preparations were quantified by micro-BCA assay and analyzed by SDS-PAGE and silver staining for purity. Western blotting using semidry transfer onto Immobilon P followed standard methods detailed previously [9] with probing using archived sdAb–AP fusion proteins and visualization with DynaLight substrate with RapidGlow Enhancer (Molecular Probes, Eugene, OR) substrate.

### Virus MARSA

Hundred microliters of neutravidin at 1 μgmL^−1^ in PBS was used to coat duplicate wells of Costar white high binding ELISA plates overnight at 4 °C. After washing 3 times with PBS to brimming, the wells were filled to brimming with Bioplex buffer (PBS, 2% w/v BSA, and 0.05% Tween-20) for an hour. The block was replaced with 100 μL of 100 nM biotinylated sdAb from pecan126 preparations in Bioplex buffer for 10 min with gentle shaking. Wells were washed 3 times with PBS 0.1% Tween-20 (PBST), 2 times with PBS, and blocked with 400 μL of PBS + 2% Carnation nonfat dried milk (MPBS). Plates were mobilized to BSL-4, and dilutions of virus were made in MPBS containing 0.1% Triton X-100 and left for 5 min. The MPBS block was removed, the virus was added in 100-μL aliquots to the wells, and plates were shaken for 10 min. After aspiration and washing 3 times with PBST and 2 times with PBS, 1 μL of phage-displayed sdAb preparation in 100 μL of MPBS was applied, and the plates were shaken for 10 min. After washing as described previously, 100 μL of 1/2500 dilution of anti-M13 horse-radish peroxidase (HRP) (GE Healthcare) in MPBS was added, and plates were shaken for 10 min. After washing, signals were developed with the SuperSignal ELISA Pico chemiluminescent substrate (Thermo Fisher) with 2-s integration using a luminometer (Turner Biosystems), and the duplicates were averaged. The assay was performed once more on a different occasion to create a graph representing the average of the two plots, with maximum and minimum bars representing ± standard deviation (SD).

### Recombinant NP MARSA

Conditions, timings, and manual pipette washings were kept consistent with the virus MARSA except that the assay was performed at BSL-2, replacing the virus with recombinant NP. The experiment was performed on two different occasions, with plots representing the average and error bars representing ±SD.

### Production, purification, and gel probing of nluc-NP C-termini

Regions of NP from the amino acid 612 to the end were either amplified by PCR or obtained as gBlocks® sequences and mobilized to pENCO9 [7] *via Not*I and *Hin*dIII with a T7 promoter driving cytosolic expression of the nluc fusion and a His_6_ tag between the nluc and NP612 regions. The plasmids were mobilized to BL21(DE3)+pRARE for expression and purification *via* IMAC and SEC, along with nluc-negative control protein as described previously [8]. Proteins were quantified by UV adsorption and analyzed by SDS-PAGE and Coomassie Blue staining. Western blotting was performed as for the NP preparations although on a higher percentage gel.

### Nanoluciferase titrations

ELISA plates were coated overnight at 4 °C with 100 μL of 1 μgmL^−1^ neutravidin in PBS. Plates were washed three times with PBS and then blocked by filling to brimming with Bioplex buffer for 1 h. Hundred microliters of 100 nM sdAb proteins from pecan126 preparations was applied to duplicate wells in Bioplex buffer for 1 h. Wells were washed to brimming 3 times with PBST and 2 times with PBS, and wells were blocked with MPBS and filled to brimming for 1 h. Dilutions of nluc-NP612 proteins in MBPS were added for 1 h. After washing, the wells were developed with an injection of coelenterazine (NanoLight™ Technology, Pinetop, AZ) in lucky buffer (10 mM Tris, 1 mM EDTA, 500 mM NaCl, pH 7.4), and signals were collected using the luminometer using a 2-s integration. The experiment was repeated once for the heterologous probing and twice for the cognate probing and EC_50_ determination. Curves are the plots of the mean RLU of nluc-NP612 minus the corresponding mean of the nluc alone, with error bars representing ±SD. The EC_50_ *y* value was calculated for curves that plateaued using the equation [RLU_min_ + (RLU-_max_-RLU_min_)/2]. The corresponding *x* values were calculated using one observed point greater and one less than the y EC_50_ using the trend function in Excel and the three values averaged and presented as ±SD nM.

### Glucibody titrations

Recombinant NP in 100 μL of PBS at 1 μgmL^−1^ was used to coat duplicate wells of Costar white ELISA plates at 4 °C. Plates were washed three times with PBS, and each well was blocked to brimming with MPBS for an hour. The wells were then probed with 100 μL of the sdAb–gluc fusions (glucibodies) in MBPS for 1 h. The probe was removed, and plates were washed by filling to brimming 3 times with PBST and 2 times with PBS. Signals were developed and processed as for the nluc titrations, with the final plots representing the mean of three experiments and the error bars representing ±SD.

### Crystallization, structure determination, and refinement

Automated screening for crystallization was carried out using the sitting drop vapor diffusion method with an Art Robbins Instruments Phoenix system in the X-ray Crystallography Core Laboratory at UTHSCSA. Crystals were obtained from commercial crystallization screen kits as follows: sdAb SB: concentrated to 8.0 mgmL^−1^ in 10 mM Tris pH 7.5 and 150 mM sodium chloride, mixed 1:1 with Molecular Dimensions Morpheus H8 containing 37.5% precipitant mix (2-methyl-2,4-pentanediol, polyethylene glycol [PEG] 1000, PEG 3350), 0.1 M amino acid mix (glutamate, alanine, glycine, lysine, serine), 0.1 M HEPES/MOPS pH 7.5 and grown at 4 °C; sdAb SB + SUDV NP610: concentrated to 20.2 mgmL^−1^ in 10 mM Tris pH 7.5 and 150 mM sodium chloride, mixed 1:1 with Qiagen JCSG Core-3 A4 containing 30% PEG 3000, 0.1 M CHES pH 9.5 and grown at 22 °C; sdAb SB + SUDV NP634: concentrated to 18.0 mgmL^−1^ in 10 Mm Tris pH 7.5 and 150 mM sodium chloride, mixed 1:1 with Qiagen JCSG Core-2 E4 containing 10% PEG 6000, 1.0 M lithium chloride, 0.1 M MES pH 6.0 and grown at 22 °C; sdAb ZC: concentrated to 12.3 mgmL^−1^ in 10 mM Tris pH 7.5 and 150 mM sodium chloride, mixed 1:1 with Qiagen JCSG Core-4 E8 containing 1.0 M sodium/potassium tartrate, 0.2 M lithium sulfate, 0.1 M Tris pH 7.0 and grown at 4 °C; sdAb ZC + EBOV NP634: concentrated to 11.7 mgmL^−1^ in 10 mM Tris pH 7.5 and 150 mM sodium chloride, mixed 1:1 with Microlytic MCSG-2 B8 containing 1.1 M malonic Acid, 0.15 M ammonium citrate tribasic, 0.072 M succinic acid, 0.18 M DL-malic acid, 0.24 M sodium acetate, 0.3 M sodium formate, 0.096 M ammonium tartrate dibasic, final pH 7.0 and grown at 4 °C; sdAb ZE + SUDV NP634: concentrated to 11.6 mgmL^−1^ in 10 mM Tris pH 7.5 and 150 mM sodium chloride, mixed 1:1 with Qiagen JCSG Core-1 H9 containing 0.8 M ammonium sulfate, 0.1 M citric acid, pH 3.5 and grown at 4 °C.

Crystals were transferred to undersized cryoloops and manipulated to wick off excess mother liquor before flash cooling in liquid nitrogen. X-ray diffraction data were acquired using a home source Rigaku MicroMax 007HF X-ray Generator equipped with VariMax HR and HF confocal optics and RAXIS-HTC image plate detectors and at the Advanced Photon Source beamlines 24-ID-C and 24-ID-E (Argonne, IL). Diffraction data were integrated and scaled using X-ray detector software (XDS) [37]. The structure of sdAb SB + SUDV NP610 was determined by the molecular replacement method implemented in MR_ROSETTA [38] using our three anti-MARV NP sdAbs in a composite search model ensemble (Protein Databank entries 6APO, 6APQ, and 4W2P [7]). The remaining NP fragment in the asymmetric unit was autotraced using PHENIX [39], followed by manual rebuilding. All other structures were determined using the resulting sdAb SB + SUDV NP610 coordinates for search models. Coordinates were refined using PHENIX, including simulated annealing with torsion angle dynamics, and alternated with manual rebuilding using COOT [40]. The sdAb ZC and sdAb ZE + SUDV NP634 data initially produced lower quality maps than would be expected for the resolution limits used for the original data processing. On further examination, strong anisotropy was identified by the UCLA-DOE Diffraction Anisotropy Server [41] and the STARANISO Server [42]. STARANISO was used to perform ellipsoidal truncation and scaling and the subsequent data sets were used for refinement with anisotropic scaling turned off in PHENIX. The diffraction limits for the sdAb ZC were 1.49 Å (best) in direction 0.93a* + 0.36b* + 0.29 + 0.02c* and 2.31 Å (worst) in direction 0.29a* + 0.29b* + 0.92c*. The highest resolution bin with spherical data completeness above 70% was 1.95–1.89 Å. The diffraction limits for sdAb ZE + SUDV NP634 were 1.93 Å (best) in direction 0.62a* + 0.79c* and 3.02 Å (worst) in direction 0.45a* + 0.82b* + 0.36c*. The highest resolution bin with spherical data completeness above 70% was 2.63–2.55 Å. TLS refinement [43] was used after individual *B*-factor refinement. For high-resolution data sets with suitable data-to-parameter ratios, individual anisotropic *B*-factors were refined. X-ray sources, data collection, and refinement statistics are shown in Table 1. Coordinates and structure factors have been deposited in the Protein Data Bank with accession numbers 6U50, 6U51, 6U52, 6U53, 6U54, and 6U55.

## Supplementary Material

1

## Figures and Tables

**Table 1.** Data collection and refinement statistics.

PDB code	sdAb SB	sdAb SB + SUDV NP610	sdAb SB + SUDV NP634	sdAb ZC^a^	sdAb ZC + EBOV NP634	sdAb ZE + SUDV NP634^a^
	6U50	6U51	6U52	6U53	6U54	6U55
**Data collection**						
X-ray source	UTHSCSA X-ray	Advanced Photon	UTHSCSA X-ray	Advanced Photon	Advanced Photon	Advanced Photon
	Crystallography	Source 24-ID-E	Crystallography	Source 24-ID-C	Source 24-ID-E	Source 24-ID-E
	Core Laboratory		Core Laboratory			
Space group	*P*3_1_21	*P*6_1_22	*C*2	*P*4_1_2_1_2	*P*4_3_2_1_2	*C*2
Cell dimensions						
*a*, *b*, *c* (Å)	47.8, 47.8, 85.4	117.9, 117.9, 145.0	174.0, 45.0, 66.4	78.40, 78.40, 37.78	87.4, 87.4, 76.9	93.4, 28.3, 86.6
α, β, γ, (°)	90, 90, 120	90, 90, 120	90, 105.9, 90	90, 90, 90	90, 90, 90	90, 114.5, 90
Wavelength (Å)	1.54178	0.97918	1.54178	0.97910	0.97918	0.97918
Resolution (Å)	42.69–1.60 (1.68–1.60)^b^	145.00–2.52 (2.66–2.52)	45.14–1.90 (2.00–1.90)	31.24–1.49 (1.58–1.49)	57.71–1.60 (1.69–1.60)	78.79–1.93 (2.04–1.93)
*R*_meas_	0.142 (1.82)	0.195 (2.23)	0.082 (1.57)	0.043 (1.97)	0.054 (1.89)	0.088 (1.69)
*R*_pim_	0.045 (0.605)	0.057 (0.660)	0.044 (0.849)	0.012 (0.555)	0.025 (0.902)	0.032 (0.685)
*CC*_1/2_	0.37	0.38	0.47	0.56	0.50	0.56
Mean *I/*σ*I*	12.1 (1.3)	12.1 (1.0)	9.5 (0.9)	23.3 (1.4)	13.5 (0.8)	10.5 (1.0)
% completeness (spherical)	99.4 (96.2)	99.5 (97.0)	96.9 (99.8)	68.3 (21.7)	96.2 (98.9)	65.3 (20.9)
% completeness (ellipsoidal)	—	—	—	94.3 (79.2)	—	90.0 (82.1)
Redundancy	9.7 (8.5)	10.4 (9.7)	3.3 (3.3)	12.3 (12.3)	4.2 (4.2)	6.4 (5.6)
Wilson value (Å^2^)	17.6	49.3	34.3	29.2	28.5	44.1
**Refinement**						
Resolution (Å)	37.27–1.60	58.94–2.52	23.32–1.90	31.24–1.49	57.71–1.60	46.26–1.93
No. of reflections	15,516	20,625	38,041	13,503	37,979	10,462
*R*_work_/*R*_free_	0.169/0.214	0.208/0.250	0.198/0.243	0.188/0.249	0.173/0.215	0.193/0.263
No. of atoms						
Protein	872	3322	3411	1081	1698	1717
Ion/solvent	8 (MPD)	—	5 (CI^−^)	—	—	—
Water	86	23	266	66	203	25
B-factors (Å^2^)						
Protein	24.5	57.7	46.9	35.3	38.9	55.3
Ion/solvent	35.2	—	52.4	—	—	—
Water	34.0	50.5	50.8	44.4	46.0	49.8
RMS deviations						
Bond lengths (Å)	0.009	0.004	0.008	0.009	0.009	0.008
Bond angles (°)	0.961	0.832	0.948	1.127	0.859	1.042
Ramachandran plot						
Favored (%)	98.2	97.8	98.0	95.8	99.0	97.7
Allowed (%)	1.8	2.2	2.0	3.4	1.0	2.3
Outliers (%)	0.0	0.0	0.0	0.8	0.0	0.0

RMS, root-mean-square; sdAb, single-domain antibody; EBOV, *Zaire ebolavirus*; SUDV, *Sudan ebolavirus*; MPD, 2-methyl-2,4-pentanediol.

a Data collection statistics for sdAb ZC and sdAb ZE + SUDV NP634 data are shown following ellipsoidal truncation and scaling performed using STARANISO.

b Highest resolution shells are shown in parentheses.
