# Supplementary material for: Paratope Duality and Gullying are Among the Atypical Recognition Mechanisms Used by a Trio of Nanobodies to Differentiate Ebolavirus Nucleoproteins
Source: J Mol Biol. Author manuscript; Available in PMC 2020 Dec 6. (PMC6990103; doi:10.1016/j.jmb.2019.10.005)
Supplement: 1 [file NIHMS1544227-supplement-1.pdf]

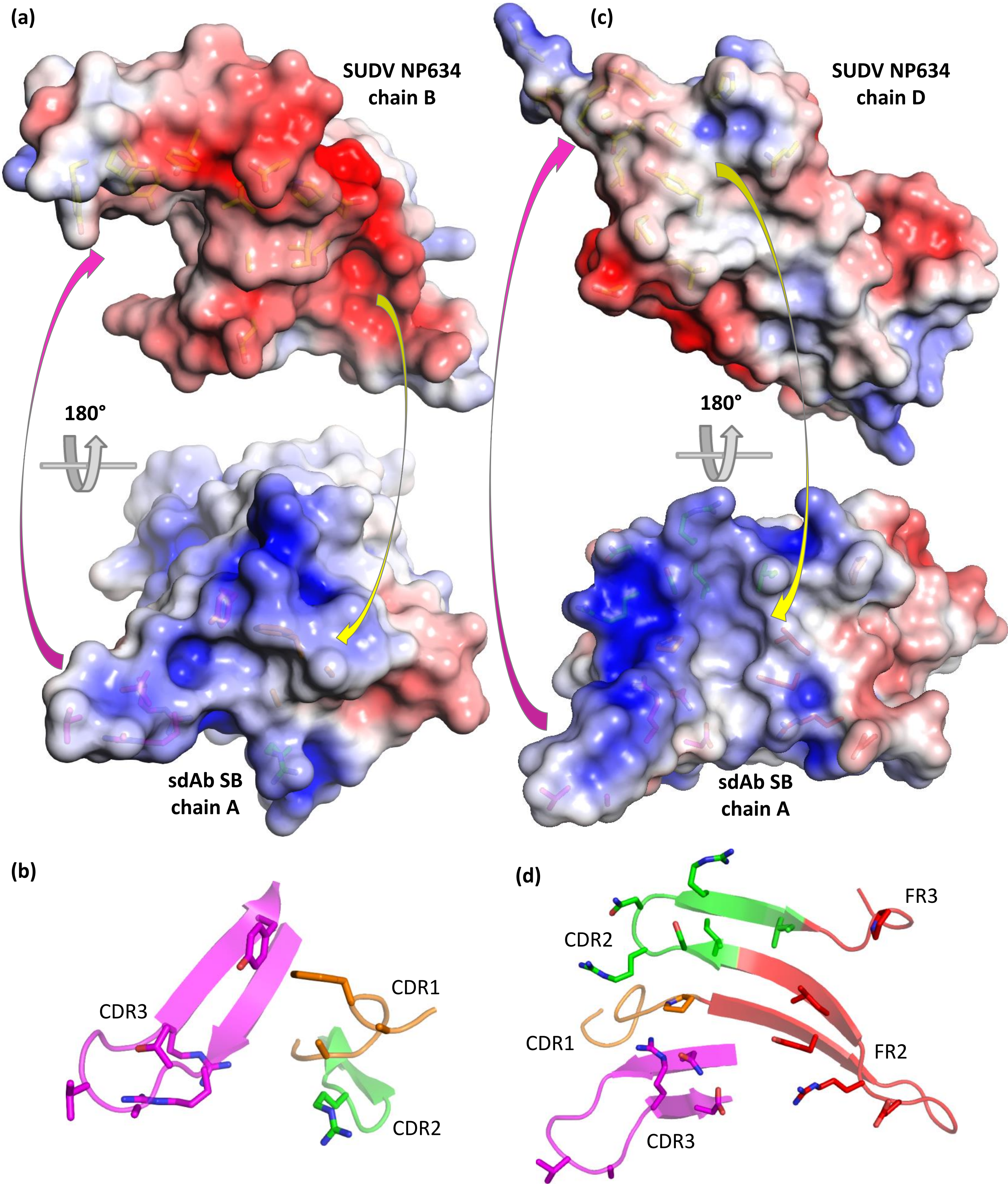

(a)
SUDV NP634
chain B
180°
sdAb SB
chain A
(c)
SUDV NP634
chain D
180°
sdAb SB
chain A
(b)
CDR3
CDR1
CDR2
(d)
FR3
CDR2
CDR1
FR2
CDR3

Supplementary Information Fig. 1. Engagement of SUDV NP634 by sdAb SB. **(a)** Top is the antibody's eye view of the epitope on chain B of NP634 while below is the NP eye's view of the sdAb paratope. Arrows indicate some of the major complementarities that are visible. **(b)** SdAb SB bound CDRs and FR2, aligned with the NP634 eye's view. **(c)** Top is the antibody's eye view of the epitope on chain D of NP634 while below is the NP eye's view of the sdAb paratope. Arrows indicate some of the major complementarities that are visible. **(d)** SdAb SB bound CDRs, FR2 and FR3, aligned with the NP634 eye's view as cartoons.
